# Functional Characterization of *Hevea brasiliensis CRT/DRE Binding Factor 1* Gene Revealed Regulation Potential in the CBF Pathway of Tropical Perennial Tree

**DOI:** 10.1371/journal.pone.0137634

**Published:** 2015-09-11

**Authors:** Han Cheng, Haibin Cai, Haitian Fu, Zewei An, Jialin Fang, Yanshi Hu, Dianjing Guo, Huasun Huang

**Affiliations:** 1 Key Laboratory of Rubber Biology, Ministry of Agriculture, Rubber Research Institute, Chinese Academy of Tropical Agricultural Science, Danzhou, Hainan, P.R. China; 2 Guangxi Subtropical Crops Research Institute, Nanning, Guangxi, P.R. China; 3 School of Life Science and the State Key Laboratory of Agrobiotechnology, The Chinese University of Hong Kong, Shatin, N.T., Hong Kong SAR, PR China; RIKEN Center for Sustainable Resource Science, JAPAN

## Abstract

Rubber trees (*Hevea brasiliensis*) are susceptible to low temperature and therefore are only planted in the tropical regions. In the past few decades, although rubber trees have been successfully planted in the northern margin of tropical area in China, they suffered from cold injury during the winter. To understand the physiological response under cold stress, we isolated a C-repeat binding factor 1 (*CBF1*) gene from the rubber tree. This gene (*HbCBF1)* was found to respond to cold stress but not drought or ABA stress. The corresponding HbCBF1 protein showed CRT/DRE binding activity in gel shift experiment. To further characterize its molecular function, the *HbCBF1* gene was overexpressed in *Arabidopsis*. The *HbCBF1* over expression (OE) line showed enhanced cold resistance and relatively slow dehydration, and the expression of *Arabidopsis* CBF pathway downstream target genes, e.g. *AtCOR15a* and *AtRD29a*, were significantly activated under non-acclimation condition. These data suggest *HbCBF1* gene is a functional member of the CBF gene family, and may play important regulation function in rubber tree.

## Introduction

Temperature determines the geographic distribution of plants in the world. Plants in the temperate regions have the ability to enhance their freezing tolerance by a process called cold acclimation, during which the plants adjust their metabolism to cold temperature and acquire enhanced freezing tolerance after being exposed to nonfreezing cold temperatures [1]. The expressions of certain genes are changed at the transcriptional level during cold acclimation [2–7], and these altered genes belong to several cold signal pathways and often interact to constitute a crosstalk gene network, which have been reviewed by several authors in recent years [1,8–14]. Briefly, two types of cold acclimation pathways are found in plants: ABA dependent and ABA independent pathway [15]. ABA dependent pathway requires the accumulation of ABA and the activation of several transcription factors, such as ABFs [16,17], MYBs and MYCs [18,19]. The transcription factors then bind with ABRE, MYBRS, and MYVRS cis-elements, and activate functional genes, such as *RD22* and *RD29B*. ABA independent pathway does not require ABA for gene activation. The most intensively studied ABA independent pathway is ICE1-CBF regulon, in which the ICE1 (INDUCER OF CBF EXPRESSION1) proteins is activated by secondary signal during cold stress. The activated ICE1 then induces the expression of CBF transcription factors [10,20,21], which bind with CRT/DRE motif [22,23]and regulate the expression of COR genes that confer freeze tolerance. CBF pathway is conserved in both cold acclimated plants and non acclimated plants. Until now CBF and its homologs have been cloned in more than fifty plant species[24], such as *Arabidopsis*, maize, grape, eucalyptus, citrus, wheat, barley, and tomato [24–30] *et al*.

Rubber trees (*Hevea brasiliensis*) originate from the Amazon rain forest and they are very susceptible to low temperature. Traditionally, rubber trees are planted in the tropical regions and are not tolerant to cold stress. Since 1950s, rubber trees began to be planted in south China on a large-scale. Nowadays, rubber tree plantation acreage has reached 1.13 million *ha* in China. However, cold stress has been one of the most influencing factors for rubber plantation in China. In the past six decades, rubber trees have demonstrated to be very sensitive to chilling stress and they could be killed by above freezing temperature in weeks. In the winter of 1954/1955, 95% of planted young rubber trees were injured severely in Guangxi province, and the percentage in Guangdong province is 80%. In 1975/1976, about 61.6% tapped rubber trees were damaged severely in Guangdong, Yunnan, Guangxi and Fujian province [31]. In the years of 2007/2008, 100% of the tapped trees were injured in the east of Yunnan province [32], and more than 70% suffered from cold in the mid and west-north regions in Hainan province [33].

In the past decades, the study on cold stress of rubber tree was mainly focused on breeding cold resistant clones and alleviating injury after cold stress. Several cold resistant rubber tree clones were bred, such as 93–114, GT1, Yunyan 77–4 *et al*. However, studies on the physiological changes during cold stress and the mechanisms of cold resistance were limited. As CBF pathway is the most important and yet less well studied pathway in non-cold acclimation plant, we wonder if the CBF regulon is functionally normal in this cold sensitive woody perennial. In this study, the *Hevea brasiliensis CBF1* gene was cloned and functionally characterized, and its role in rubber tree cold stress was discussed.

## Materials and Methods

### Manipulation of DNA, RNA Isolation, and Expression Analysis

The rubber tree DNA and RNA isolation was carried out as described previously [34]. The *Arabidopsis* total RNA was extracted using Qiagen RNeasy Plant Mini Kit (Qiagen, USA). The RNA samples used for RT-PCR analysis were treated with DNase I to remove possible DNA contamination. Two microgram total RNA was used for the each detection. The first-strand cDNA was synthesized with SuperScript III (Invitrogen) according the instructions. Quantitative RT-PCR was carried out using IQ SYBR green supermix (Bio-Rad, USA) with gene specific primers listed in S1 Table. PCR was performed on a Bio-Rad Iq5 RT PCR instrument using following program: 95°C for 3 min, followed by 40 cycles of 95°C for 15 sec, 60°C for 30 sec and 72°C for 30 sec.

For Northern blotting, *HbCBF1* probe was prepared from full length cDNA with *DIG* Northern Starter *Kit* (Roche, USA). Twenty micrograms of total RNA was run on 1.5% denature agarose gels and the blot was then transferred onto Amersham Hybond^TM^-N^+^ nylon membranes (GE Healthcare, USA). The hybridization and detection was carried out according to the manufacturer’s instructions.

### Bioinformatic and phylogenetic analysis

Multiple sequence alignments were performed using ClustalW2 at the EBI ClustalW server (http://www.ebi.ac.uk/Tools/msa/clustalw2/) using default parameters [35]. The CDS was predicted using Bioedit software and confirmed using BLASTP program at NCBI BLAST server (http://blast.ncbi.nlm.nih.gov/Blast.cgi). The protein secondary domains were predicted with Interproscan program [36] at EBI server (http://www.ebi.ac.uk/Tools/pfa/iprscan5/). Mega software (version 6.06) was used for phylogenetic analysis [37]. The phylogenetic tree was consctructed using the neighbor-joining method with bootstrap test. The default parameters were used for the construction.

### Southern blotting

For Southern blotting analysis, 40 μg rubber tree genome DNA was digested with *Eco*RI, *Pst*I, *XbaI* and *HindIII* respectively. After resolved on 0.8% agarose gel, the blot was transferred onto Amersham Hybond-N^+^ nylon membrane (GE Healthcare, USA) and cross-linked under 0.12 J/cm^2^ UV irradiation. Then the blot was hybridized at 42°C with Digoxigenin labeled probe for 12h. After 2 stringent washes at 68°C, the signal was detected using DIG Nucleic Acid Detection Kit (Roche, USA).

### Electrophoretic Mobility Shift Assay

To prepare recombinant HbCBF1 protein, the CDS region of *HbCBF1* gene was amplified using primers 5’-CCCTCGGATCCATGGATGTTTTC-3’ AND 5’-CAAGCTATGCGGCCGCTCTTAT-3’, and sub-cloned into pGEX-6p-1 plasmid. The GST-HbCBF1 fusion construct was transformed into BL21(DE3)*pLysS* cells, and fusion protein was prepared with 0.1 mmol/L IPTG induction for 3 h. Total soluble protein was prepared and used for mobility shift analysis.

Electrophoretic mobility shift assay was carried out as described [38]. Briefly, 4 *μ*g of GST-HbCBF1 fusion protein was incubated with 0.5 μg double strand fish sperm DNA (Roche, USA). Cold probe was used as competitor (125-fold molar excess) or as indicated elsewhere. The binding buffer consist of 10 mM Tris-HCl, pH 7.5, 100 mM KCl, 10% (v/v) glycerol, 1 mM dithiothreitol, 0.1% (v/v) nonidet P-40 and 1 mM spermidine. The mixture was incubated for 10 min at 25°C, then 1 ng labelled probe was added. After further 20 min incubation, the reaction was loaded on 6% polyacrylamide gels containing 44.5 mM Tris, 44.5 mM borate, 1 mM EDTA, and 5% glycerol. The blot was then semi-dry transferred to Amersham Hybond-N+ nylon membrane and cross-linked under 0.12 J/cm2 UV irradiation. Digoxigenin was detected using DIG Nucleic Acid Detection Kit (Roche, USA).

The 5'-Digoxigenin labeled probe and non-labeled oligonucleotides were synthesized (Takara, Dalian, China) and sequences were as follows: COR15a, TTTCATGGCCGACCTGCTTTTTT, COR15b, CTGATGGCCGACCTCTTTTTT, RD29a, ATATACTACCGACATGAGTTCT, RD29b, CTACCGACATGAGTTCCAAAAAA, M1, TTTCAAGAATTCACTGCTTTTTT, M2, TTTCATGGTATGTCTGCTTTTTT, M3, TTTCATGGAATCACTGCTTTTTT.

### Plant Material, Preparation of Transgenic Plants

One year old rubber tree clone 93–114 was grown in plastic bag and cultured in a greenhouse at the temperature of 27°C with 45% humidity. *Arabidopsis thaliana* ecotype Columbia-0 (*col-0)* was used as wild-type in this study. Seeds were surface sterilized and sowed in pots (10cm×10cm×10cm) containing a mixture of soil and vermiculite (3:1). After 4d imbibitions at 4°C, the plates and Arabaskets were transferred to the growth chamber at a constant temperature of 20°C under 16 h light (125 μmol m^–2^ s^–1^)/8 h dark cycles.

To generate *HbCBF1* OE lines, the coding region of *HbCBF1* gene was amplified using primers 5’-CCTCTAGACTATGGATGTTTTCCCT-3’ and 5’-CCCGAGCTCTTATATAGAAAAACTCCA-3’. The CDS fragment was then cloned into pBI121 plasmid. *Arabidopsis* ecotype *col-0* plants were transformed according to the floral dip method [39] using *Agrobacterium tumefaciens* strain GV3101(*pMP90*).

### Cold, drought stress and ABA treatment

Cold acclimation was carried out by transferring seedlings into 4°C cold chamber for 5 days. For stress resistance test, two weeks old transgenic *Arabidopsis* seedlings were subjected to freezing stress by being exposed to -6°C for 18 hours. Thirty to fifty plants were subjected to each treatment in three replicates. All the *Arabidopsis* plants were restored to normal growth conditions after stress treatments. The number of survived plants was counted. The electrolyte leakage analysis was performed as described [40]. Briefly, electrolyte leakage was measured using leaves from 2 weeks old seedlings by frozen to -7°C at a refrigerating rate of 1°C per h from -1°C with a A28F Thermo Fisher temperature-controlled water bath (Thermo Scientific, USA). For dehydration rate calculation, leaves were detached from 2 weeks old seedlings and placed on dry filter paper in a drying chamber. The samples were weighed at each 30 min interval and the dehydration rates were calculated as the ration of the remaining weight to initial weight at each time point.

For cold treatment in rubber tree, the seedlings were transferred to a 4°C cold culture room. Drought was carried out by detaching the leaf from the seedlings and keeping on the filter paper at room temperature. For ABA treatment, 100 μmol/L ABA solution was sprayed onto the seedlings until the liquid dropped from the leaf. Then the seedlings were covered with a plastic bag. The leaf sample was collected at indicated time points and stored in liquid nitrogen immediately.

### Statistical analysis

All results were presented as mean ± standard error of mean of three biological replicates for qPCR, or five biological replicates for stress study. The statistical significance analysis was performed by using the Student’s *t*-test, and *p* < 0.05 was recognized as significant.

## Results

### Isolation and Characterization of *HbCBF1* gene

**Fig 1:**
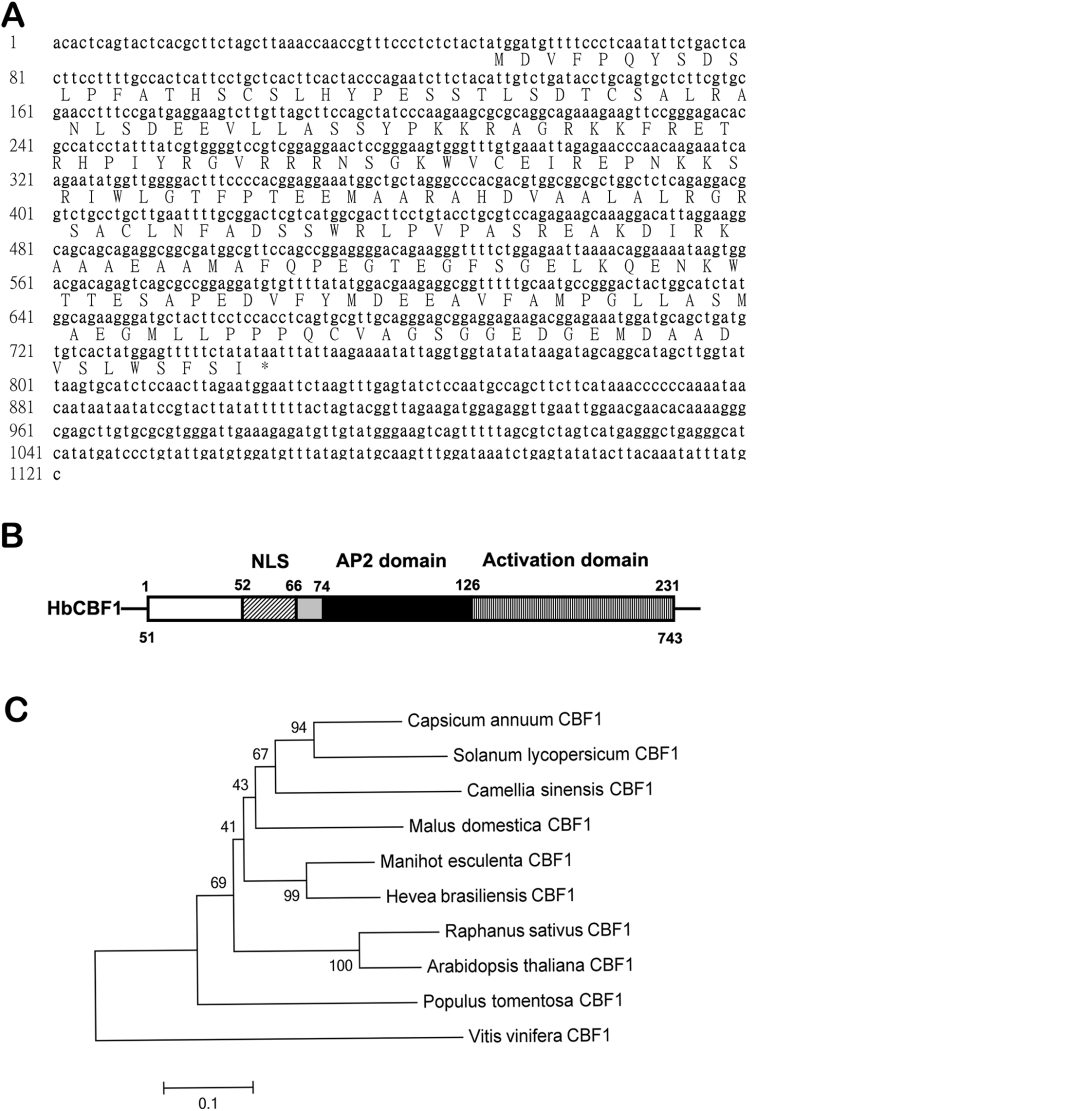
(A) Full length cDNA sequence of rubber tree *C-repeat binding factor 1* (*HbCBF1*) gene and deduced encoding peptides. (B) Conservative domains distribution in HbCBF1 protein. NLS, nuclear localization signal. (C) Phylogenetic tree showing similarity of the deduced *Hevea brasiliensis* CBF1 protein sequence with those described in other plants. Sequences of each species were downloaded from Genbank by the following accession numbers: *Capsicum annuum* CBF1, AAZ22480.1; *Solanum lycopersicum* CBF1, AAS77820.1; *Camellia sinensis* CBF1, AFN93974.1; *Malus domestica* CBF1, AAZ20446.1; *Manihot esculenta* CBF1, AFB83707.1; *Raphanus sativus* CBF1, ACX48435.1; *Arabidopsis thaliana* CBF1, AEE85066.1; *Populus tomentosa* CBF1, ABC79626.1; *Vitis vinifera* CBF1, AEM37861.1. The phylogenetic tree was constructed using a Mega 6.06 software.

To isolate CBF-like genes from *Hevea brasiliensis*, degenerated primers C007 and C008 (S1 Table) were used for RT-PCR analysis. The amplified 430-bp fragment was cloned and sequenced, and revealed to be related to AP2 gene family. RACE was then used to isolate the full length cDNA. Sequencing results revealed it was 1121 bp in length, containing an ORF of 231 amino acid residues (Fig 1A). The deduced HbCBF1 protein contains a conserved DNA binding domain (66–126 amino acid residues) and a positively charged NLS cluster “KKRAGRKKFRETRHPIYRGVRRR” (52 to 74 amino acid residues, Fig 1B). This structure was very similar to the CBFs in other plants. Genomic PCR produced the same sequence of cDNA, showing no intron in this gene. The candidate gene was named as *HbCBF1* and its complete sequence was deposited into the GenBank (Accession: AY960212). The phenogram generated by the MEGA analysis (Fig 1C) demonstrated that HbCBF1 is mostly similar to the *Manihot esculenta* (cassava) CBF1 (accession no. AFB83707.1), which also belongs to the family of *Euphorbiaceae*.

**Fig 2:**
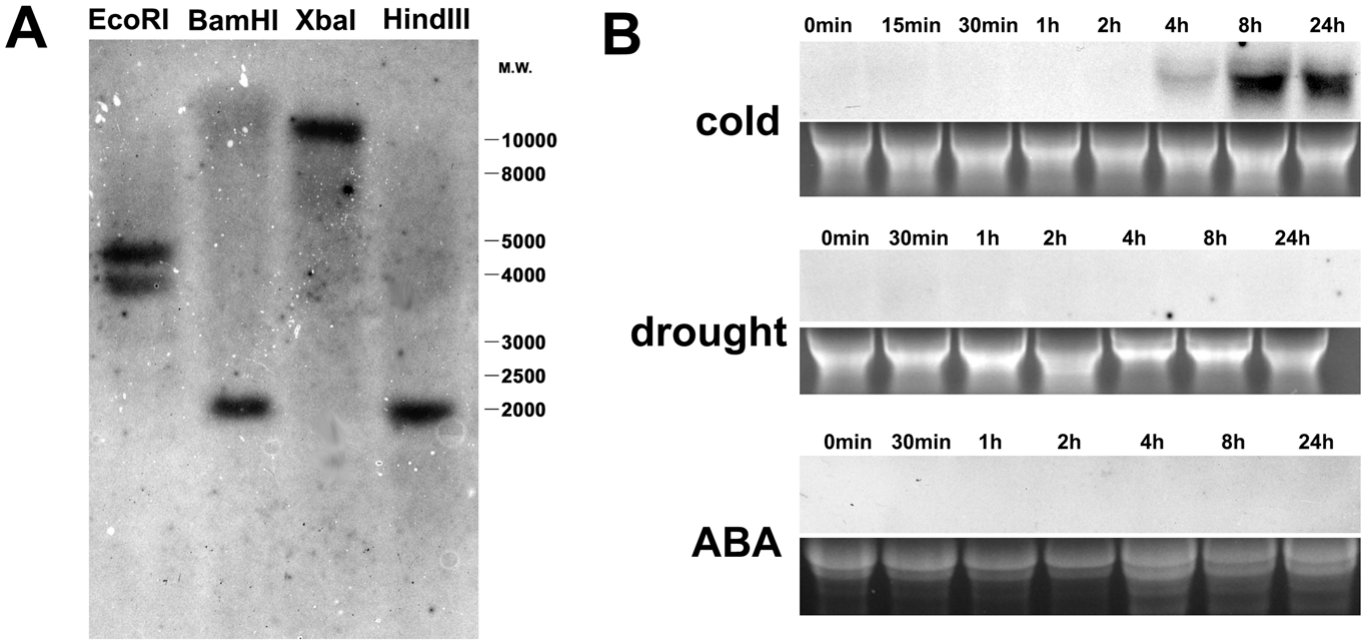
Characterization of *HbCBF1* gene. (A) Southern blotting analysis of *HbCBF1* gene in *Hevea brasiliensis* genome. (B) *HbCBF1* expression in response to cold, drought stress or ABA treatment. Twenty microgram total RNA from the leaves of the seedlings treated with cold, drought or ABA for the indicated time were used for Northern blotting analysis. *HbCBF1* full length cDNA was used as probe. EtBr-stained rRNA was used as an internal standard to monitor equal loading of total RNA.

Rubber tree genomic DNA was digested with several restrictive enzymes and transferred onto nylon membrane. The blot was hybridized at high stringency using the probe prepared from *HbCBF1* full-length cDNA (Fig 2A). Only one band was detected for *Bam*HI, *Xba*I and *Hin*dIII digestion, while the *Eco*RI digestion produced 2 bands (*HbCBF1* gene contains one *Eco*RI restrictive site). The blotting results indicated that the *HbCBF1* is not replicated in the *Hevea brasiliensis* genome.

### *HbCBF1* expression shows response to cold but not drought or ABA treatment

The gene expression profiles toward stresses and ABA were analyzed using Northern blotting. At first, we tested the effectiveness of the treatments in rubber tree using a *RD22* homolog gene which showed response to cold, drought or ABA treatments in rubber tree (S1 Fig). The *HbRD22* expression pattern in response to various stress or ABA treatment are similar to the homolog *RD22* gene in *Arabidopsis* [18], showing that the treatments are effective to induce stress related gene expression in rubber tree.

The *HbCBF1* transcript was detected at 4 h after exposure to cold stress, and the gene expression reached its highest level at 8 h, and then began to decrease after 24h (Fig 2B). This pattern was quite different from that of any other reported *DREB1*/*CBF* genes, which were often induced in 15 min under cold treatment [41–43]. When subjected to drought stress or ABA treatment, no gene expression was detected in 24 h. However, we cannot exclude the possibility of induction of *HbCBF1* by prolonged drought stress and ABA treatment. Similarly, the tomato *LeCBF1* gene also showed no response to drought or ABA either [44].

### Gel mobility shift assay revealed CRT/DRE binding activity of HbCBF1 protein

**Fig 3:**
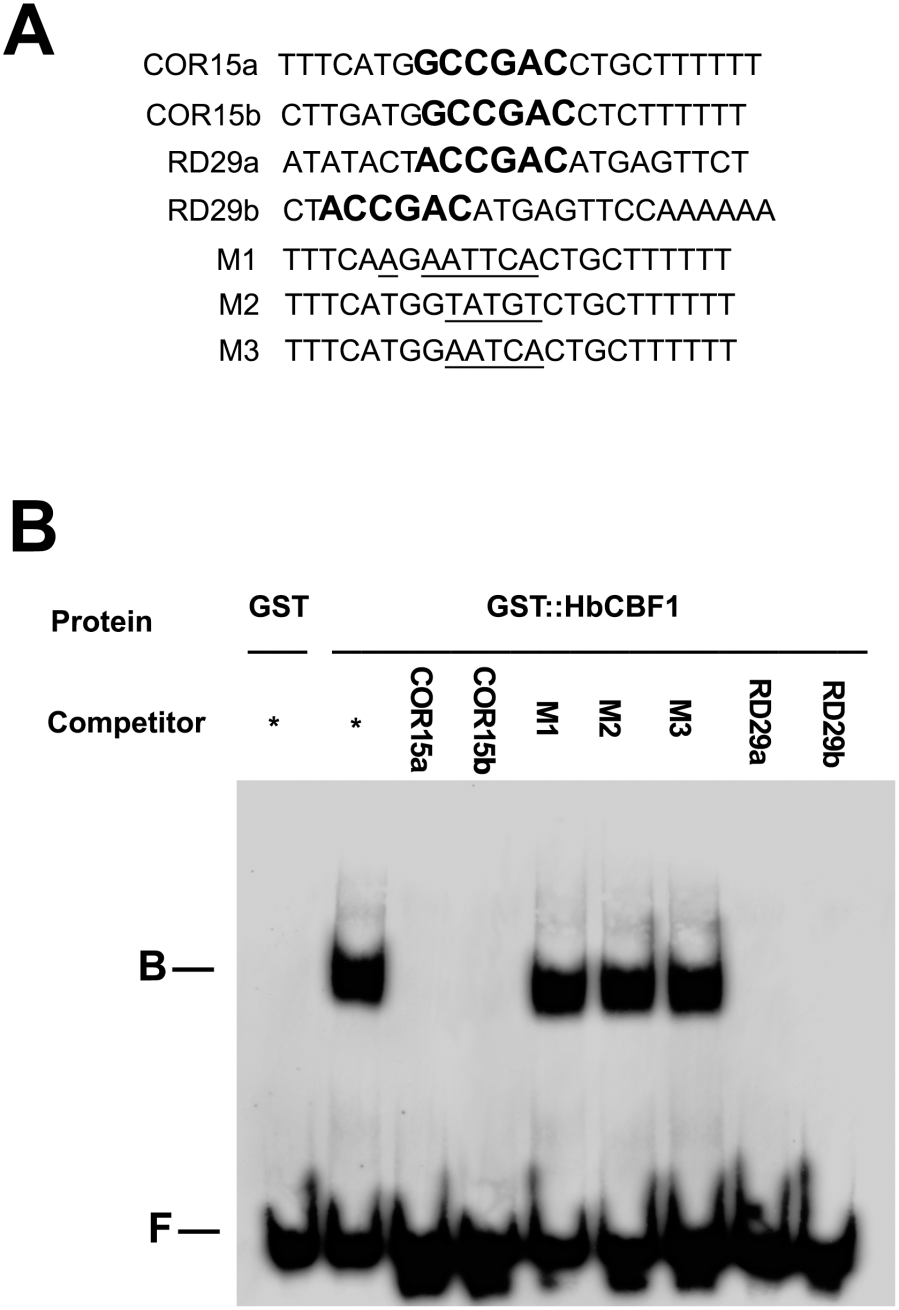
Gel mobility shift assay of recombinant HbCBF1 protein indicated CRT/DRE binding activity. **(A)** Probes sequences used in this analysis. Bolded nucleotides are CRT/DRE motif, while underlined nucleotides are the mutation sites. **(B)** HbCBF1 bound with CRT/DRE elements specifically *in vitro*. Four microgram total proteins of GST (lane 1 from the left) or GST::HbCBF1 (lane 2–9 from the left) were used for the binding with labeled hot COR15a probe. One hundred and twenty-five folds molar unlabelled cold competitor probes were added as indicated (labe 3–9 from the left) in each reactions. The retarded binding bands and free non-binding probe were indicated as "B" and "F" respectively.

Gel shift assay was carried out to confirm the CRT/DRE binding activity of the HbCBF1 proteins. As shown in Fig 3A, no binding band was detected for proteins from the control *E*.*coli* strain whereas the recombinant GST-HbCBF1 proteins produced unique retarded band, indicating the CRT/DRE binding activity of HbCBF1 proteins (Fig 3B). Competition experiments were conducted to check the binding specificity. One hundred and twenty-five fold excessive competition probes of *AtCOR15a*, *AtCOR15b*, *AtRD29a* and *AtRD29b* completely blocked the retarded bands, indicating the binding is sequence specific. When the competition probes were mutated at the CRT/DRE motif, the binding band could not be blocked (Fig 3B), demonstrating that HbCBF1 protein binds to RCCGAC site specifically *in vitro* [22,23].

### *Arabidopsis* overexpressing *HbCBF1* showed enhanced cold resistance

**Fig 4:**
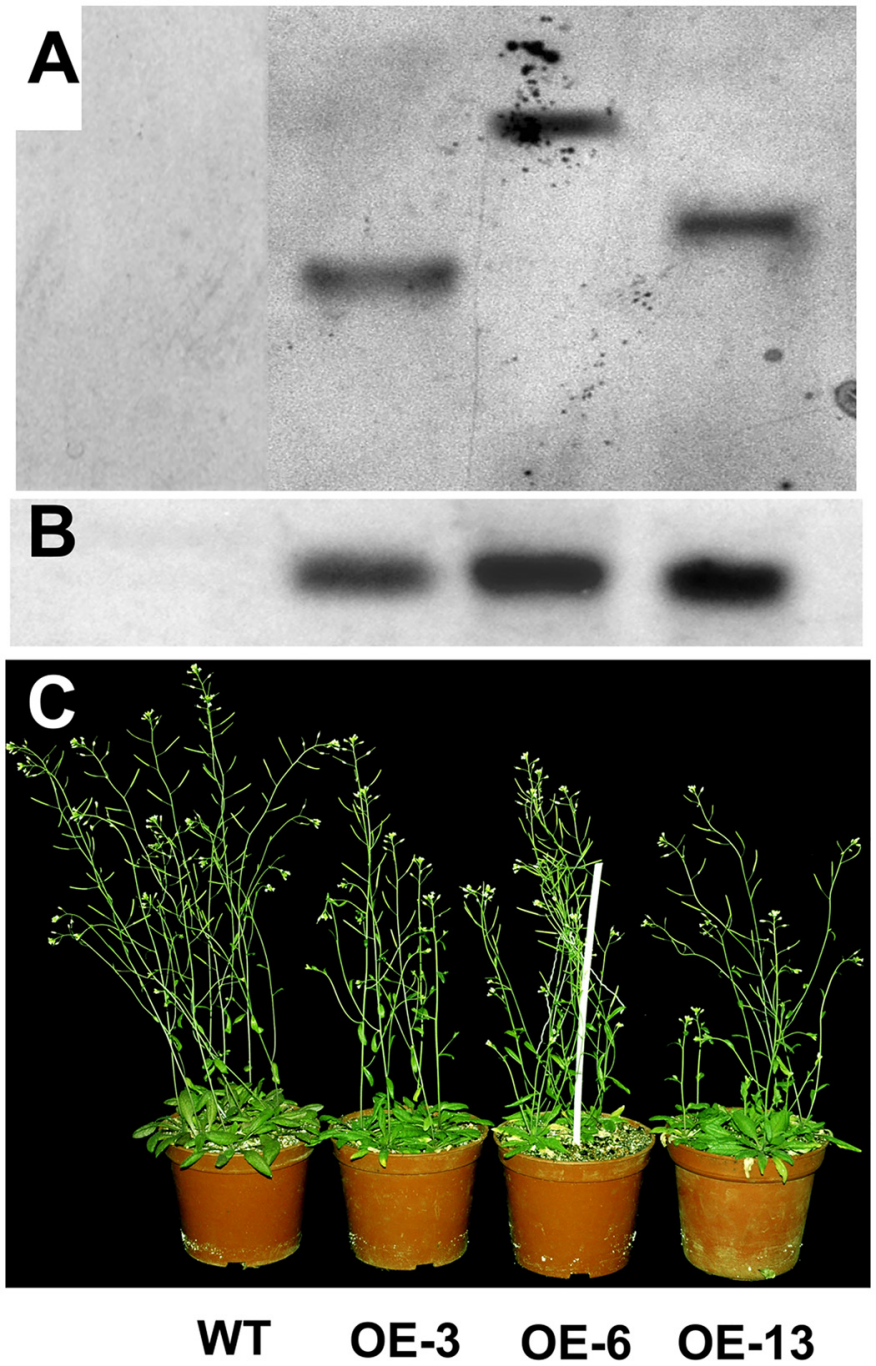
*HbCBF1* overexpression in *Arabidopsis*. (A) Southern blotting of *HbCBF1* in transgenic plants. (B) *HbCBF1* expression in OE-3, OE-6 and OE-13 lines. The gene expression was detected by Northern blotting analysis using the *HbCBF1* probe. (C) Thirty days old seedlings of *col-0*, OE-3, OE-6 and OE-13 lines showed decreased plant height.

The *HbCBF1* gene was overexpressed in *Arabidopsis* using floral dip method. Among tens of T1 transgenic lines, we selected OE-3, OE-6, and OE-13 for further analysis. These three lines harbored single copy insertion and showed high gene expression level (Fig 4A and 4B). Southern blotting was used to confirm the gene transfer. Similar to the other CBF genes, the overexpression of *HbCBF1* gene caused significant decrease of plant height in *Arabidopsis* (Fig 4C and S2 Fig).

**Fig 5:**
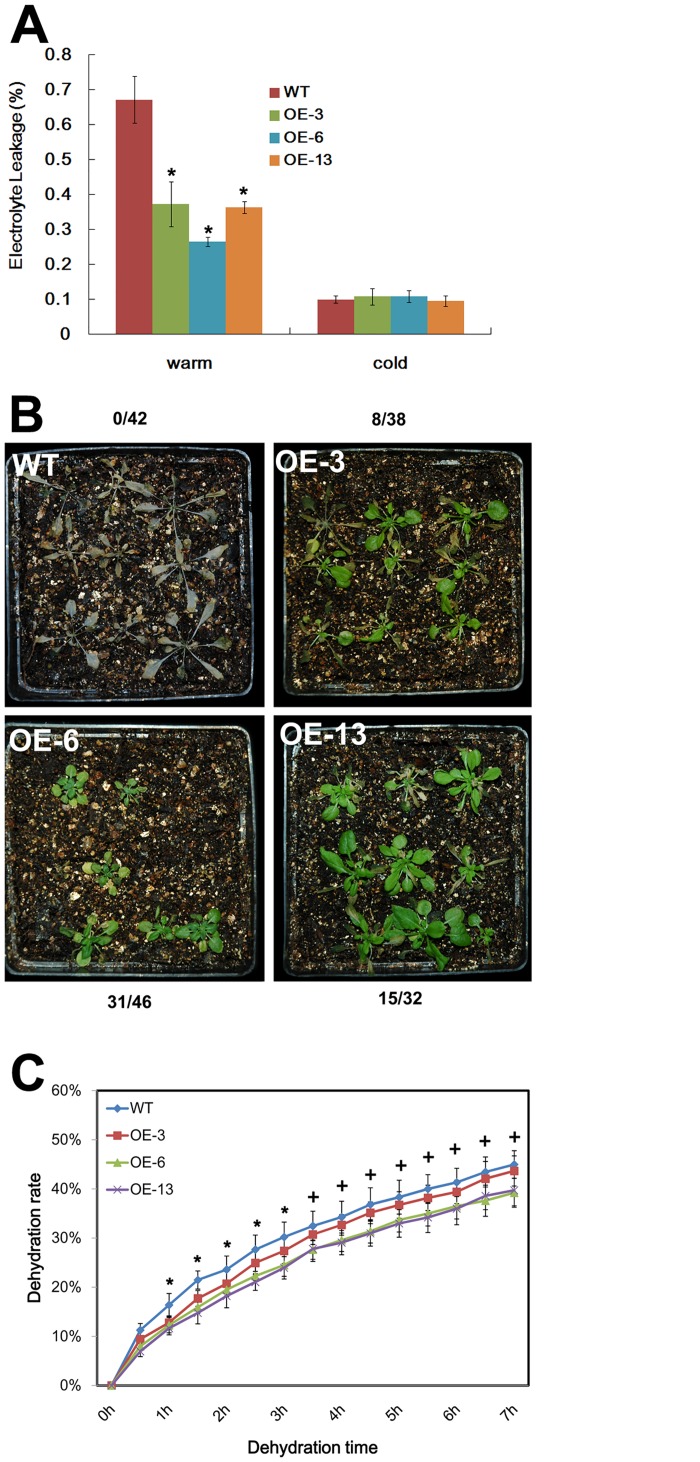
*HbCBF1* overexpression conferred cold resistance and relatively slow dehydration in *Arabidopsis*. (A) The electrolyte leakage rates of *col-0*, OE-3, OE-6 and OE-13 lines under nonacclimated (warm) or 5-days cold—acclimated conditions (cold). Electrolyte leakage was measured using leaves from two-week-old seedlings frozen to -7°C at a refrigerating rate of 1°C per h from -1°C. The star symbol (*) indicates significant difference between each individual OE line and wild type plants (WT). (B) Representative seedlings of *col-0*, OE-3, OE-6 and OE-13 lines recovered from 18 hours frozen at -6°C. The survival rates of each line were listed. (C) The dehydration rates of the detached leaves from *col-0*, OE-3, OE-6 and OE-13 seedlings. The star (*) symbol indicates significant difference between WT and each individual OE line, while plus symbols (+) shows significant difference between WT and individual OE-6 and OE-13 line. Data in A and C were presented as mean ± standard error of mean of five biological replicates. Significance was determined by Student’s *t* test at the probability levels of *P* < 0.05. Comparisons were made between the wild type plant and each individual overexpressing line. For B, totally thirty to fifty plants were tested for each line and about ten seedlings were grown in each pot.

The electrolyte leakage analysis was used to evaluate the cold resistance in the transgenic *Arabidopsis*. A significantly lower leakage rates was detected for *HbCBF1* OE lines compared to the *col-0* control. As shown in Fig 5A, the control plants had a leakage rate about 67% in non-acclimated condition, while the rates were 37%, 26% and 36% for OE-3, OE-6 and OE-13 lines respectively. The leakage rates after cold acclimation did not show significant difference. To evaluate the freezing tolerance, 2 weeks old *HbCBF1* OE plants were subjected to a low temperature of -6°C for 18 hours. After 2 days recovery, the survival rate of each line was counted. As shown in Fig 5B, all the *col-0* plants died after freezing, while the seedlings survived at the rate of 8/38, 31/46 and 15/32 for *HbCBF1* OE-3, OE-6 and OE-13 respectively (Fig 5B). These results indicated *HbCBF1* OE plants were more cold resistant than *col-0*.

Similar results were obtained when the OE plants were subjected to drought. The dehydration rates of the detached leaves were calculated. The results showed the OE-3 leaves dehydrated significantly slower than that of the wild type plants at the timeslots from 1h to 3h, while OE-6 and OE-13 dehydrated significantly slower from 1h to 7h (Fig 5C). These results implied the *HbCBF1* OE plants might loss water more slowly compared to the wild type when subjected to drought stress.

### *HbCBF1* overexpression activated COR genes in *Arabidopsis*

**Fig 6:**
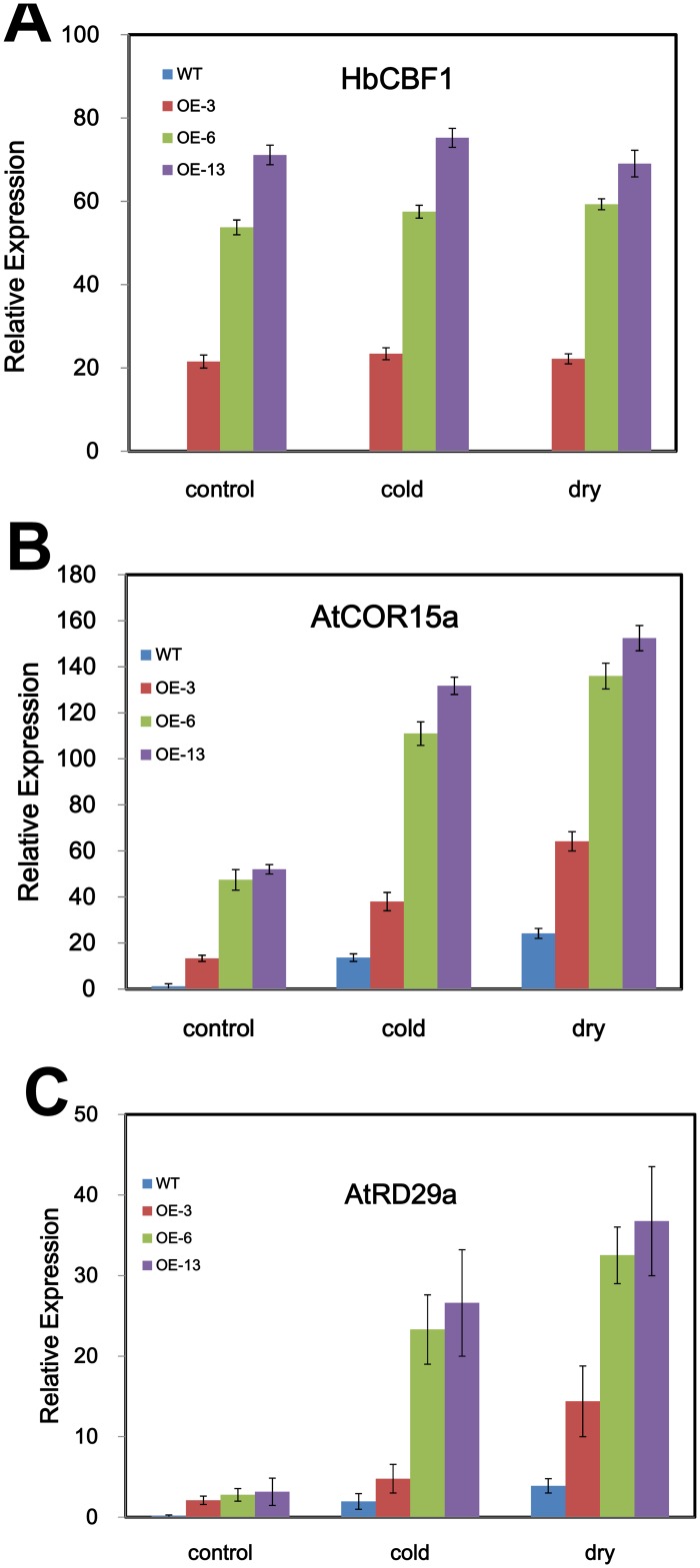
*HbCBF1* overexpression activated the expression of *COR15a* and *RD29a* genes in *Arabidopsis*. **(A)** *HbCBF1*, (B) *AtCOR15a* and (C) *AtRD29a* expression in *col-0*, OE-3, OE-6 and OE-13 plants under control condition (control), 5-day cold-acclimated (cold) or 10-days drought stress (drought). Total RNA was extracted from 2 weeks seedlings and real time PCR was performed with gene specific primers listed in S1 Table. The transcripts level was normalized to that of ACTIN 7 gene. Two microgram total RNA was used for sample and the data were presented as mean ± standard error of mean of three biological replicates. Significance was determined by Student’s *t* test at the probability levels of *P* < 0.05.

It is known that the CBF transcriptional factors regulate the downstream COR family genes. We therefore used real time PCR to check if the expression of COR genes is activated in *Arabidopsis HbCBF1* overexpression lines. In the control condition, the *COR15a* and *RD29a* transcripts were not detected or only at very low level in *col-0*. However, the expression of these two genes was significantly higher in the OE-3, OE-6 and OE-13 plants (Fig 6B and 6C). When subjected to cold or drought stress, the *COR15a* and *RD29a* transcripts increased several folds in both *col-0* and OE lines seedlings (Fig 6B and 6C). The expression of *COR6*.*6*, *COR47* and *RCI2a* were also activated in the OE-3, OE-6 and OE-13 lines. In the wild type seedlings, these genes showed no or very low level expression under control conditions (S3 Fig).

## Discussion

Rubber trees originate from tropical Amazon basin, and were introduced to South-East Asia about 150 years ago. Due to the cold susceptible trait, rubber trees were traditionally planted in a restricted region between 15° north and 15° south latitudes. In China, although farmers have planted rubber tree for more than 110 years, the acreage was still very small due to the low temperature restriction. Though some cold resistant rubber tree clones were bred and cultivated in south China on large-scale, they are still under the threaten of cold wave during winter season. Cold stress is the major restrictive environmental factor that adversely affect rubber plantation in China.

We conducted the characterization of a CBF like gene *HbCBF1* from non-acclimate woody rubber tree. This gene has the typical structural characteristics of a *CBF* gene, including a nuclear localization signal at 5' terminal, and an AP2 domain and transcription activation domain. Genome wide identification of the *Hevea brasiliensis* AP2/ERF superfamily by RNA sequencing reported 142 contigs with full AP2 domain [45]. The *HbCBF1* gene was classified into ERF III group which have 11 members. The characterization of other *CBF* like genes in this group should be helpful for understanding the CBF pathway in rubber tree.

Expression study indicated that *HbCBF1* showed responses to cold, but not drought or ABA treatment. When exposed to low temperature, *HbCBF1* gene expression was detected after 4h treatment, and reached its highest level after 8h. The expression of *HbCBF1* displays a quite slow response pattern when compared with that of *CBF1* in *Arabidopsis* [46], maize [26], *Eucalyptus* [42] and other plants, whose expression could be detected in 15 min and reached the highest level in 2–5 h. No expression could be detected until 24h after drought or ABA treatment, indicating *HbCBF1* gene did not respond to drought and ABA. These characteristics are quite different with those of *CBF1* in other species, suggesting the functional limitation of *HbCBF1*.

EMSA was performed to check the binding activity of recombinant HbCBF1 protein. Results showed that the recombinant HbCBF1 protein could bind with CRT/DRE elements from both *Arabidopsis COR15* and *RD29* genes, suggesting *HbCBF1* can potentially activate downstream *COR* genes. To further test its potential regulatory function, *HbCBF1* was overexpressed in *Arabidopsis*. In the selected OE lines, overexpression of *HbCBF1* successfully activated the expression of *AtCOR15a*, *AtRD29a* and other COR family genes in control conditions. The OE plants also showed significantly increased resistance to freezing and drought stress, which is similar to *CBF1* found in other species [25,27,29,44]. The EMSA and transgenic results suggest that HbCBF1 may potentially regulate COR functional genes in CBF pathway.

The results presented here also suggested *HbCBF1* gene possessed potential function in activating CRT/DRE targets genes. However, evidences from other non CA plant suggested that CBFs from non CA plant have smaller and less diverse regulon than that of CA plant [44,47]. Besides, less CBF target genes were found in non CA plants. Until now, only few publications reported homologous analog of *Arabidopsis* COR genes in non CA plants [47,48]. The gene structure study in *Medicago* revealed that cold acclimated *M*.*falcata* has two candidate CBF targets (*MfCAS30* and *MfCAS31*) while non cold acclimated *M*. *truncatula* has only one (*MtCAS31*). In addition, the number of CRT/DRE element in the upstream region of *CAS31/CAS30* may be a factor to determine the cold tolerance between the CA and the non CA *Medicago* species [47]. The study of CBF regulon in *Medicago* affords us lessons for the study in rubber tree which is extremely cold sensitive. The survey of *Hevea* draft genome (TBLASTN program against rubber tree genome database with thresholds of e-value less than 0.01) using *Arabidopsis* COR6.6, COR47, COR15a and RD29a protein sequences revealed the existence of two COR47 like homolog [49]. Both these two COR47 like homologs contain CRT/DRE motifs in the upstream regulation region. Besides, the searching with other known COR gene family (such as *Medicago falcate* CAS18 (Accession: AAA21185.1), *Medicago sativa* CAS15 (Accession: AAA16926.1) and CORa (Accession: AAA99833.1), *Triticum aestivum* WCOR615 (Accession: AAB18208) and WCS120 (Accession: AAA34261.2) [50]) also found one CAS18 like homolog. As COR genes are not phylogenetically related, the homologous searching with the COR gene families could not exclude the existence of rubber tree COR genes that are not related to the known CORs. However, among the known COR gene families, the number in rubber tree is quite less compared with that in *Arabidopsis* or other CA plants [50]. As *HbCBF1* has the potential to regulate downstream COR regulons, the shortage of COR family genes may be one of the reasons for the cold sensitivity of rubber tree. To verify this hypothesis, further work should be focused on functional characterization of these two COR homologs and identification of more CBF target genes in *Hevea brasiliensis*.

## Supporting Information

S1 Fig Cold, drought and ABA treatment induced the expression of *HbRD22* gene in *Hevea brasiliensis*. *Hevea* seedlings were treated with cold, drought and ABA as described in *Material and methods*. Total RNA was extracted and 20 microgram RNA of each sample was used for Northern blotting. The hybridization was performed against the probe prepared from an *HbRD22* full-length cDNA.
(TIF)

S2 Fig *HbCBF1* overexpression decreased the plant height in *Arabidopsis*. *Arabidopsis* seedlings of *col-0* and *HbCBF1* overexpression lines OE3, OE6 and OE13 were cultured as described in *Material and methods*. The seedling height was measured at 30 days old. The data were presented as mean ± standard error of mean of 15 to 20 plants. Star symbol (*) shows the significance when compared with *col-0* plants. The statistical significance analysis was performed by using the Student’s *t*-test, and *p* < 0.05 was recognized as significant.
(PDF)

S3 Fig *HbCBF1* overexpression activated the expression of *COR6*.*6*, *COR47* and *RCI2A* genes in *Arabidopsis*. *AtCOR47* **(A)**, *AtCOR6*.*6* **(B)** and *AtRCI2a* **(C)** expression in *col-0*, OE-3, OE-6 and OE-13 plants under nonacclimated (control), 5-day cold-acclimated (cold) or 10-days drought stress (drought). Data were presented as mean ± standard error of mean of three biological replicates. Significance was determined by Student’s *t* test at the probability levels of *P* < 0.05.
(PDF)

S1 Table List of oligonucleotide sequences used in this study. (DOCX)
