# Supplementary figures and images for: Functional Characterization of Hevea brasiliensis CRT/DRE Binding Factor 1 Gene Revealed Regulation Potential in the CBF Pathway of Tropical Perennial Tree
Source: PLoS One. 2015 Sep 11;10(9):e0137634. doi: 10.1371/journal.pone.0137634 (PMC4567348; doi:10.1371/journal.pone.0137634)

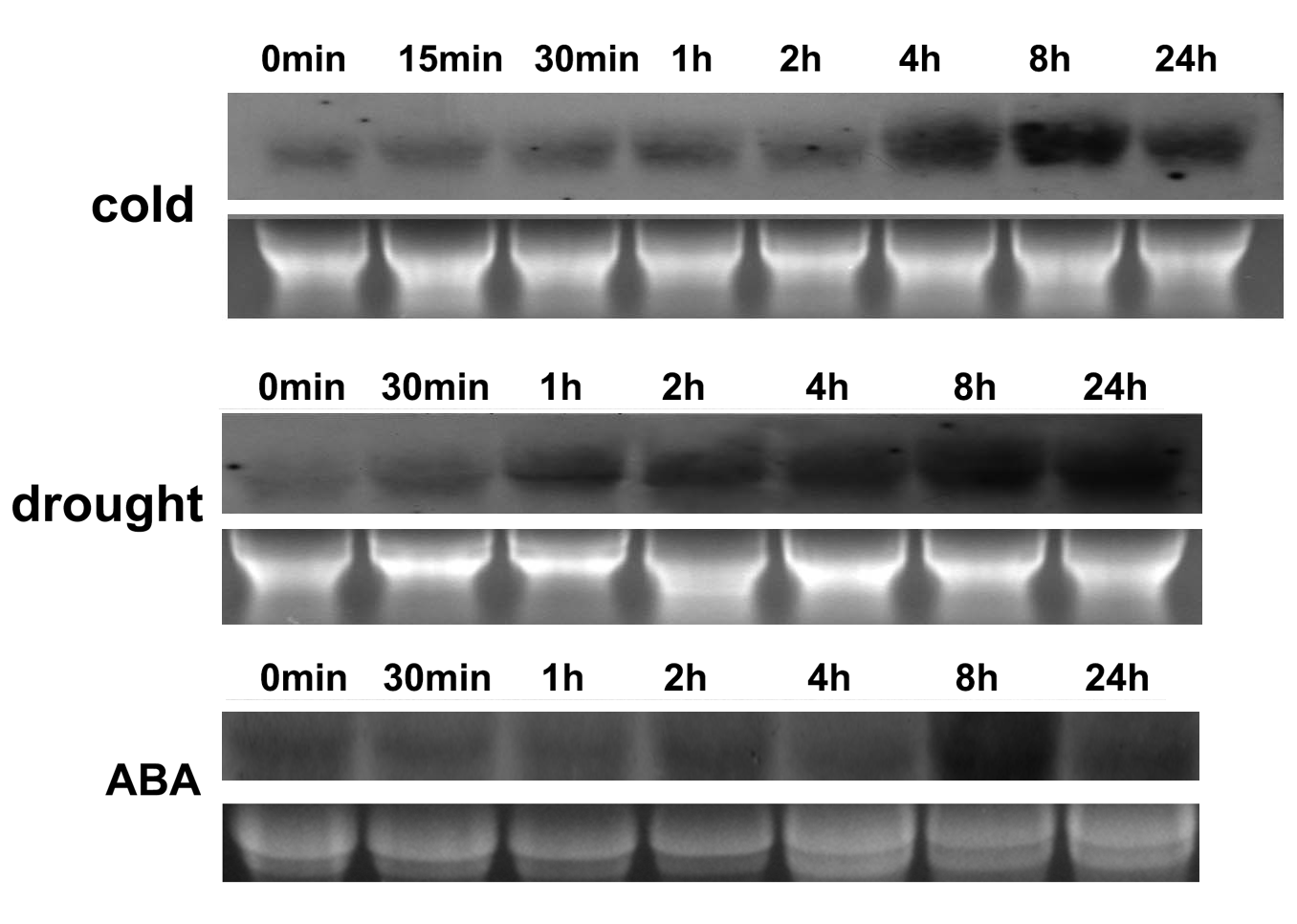

Supplement: S1 Fig — Hevea seedlings were treated with cold, drought and ABA as described in Material and methods. Total RNA was extracted and 20 microgram RNA of each sample was used for Northern blotting. The hybridization was performed against the probe prepared from an HbRD22 full-length cDNA. (TIF) [file pone.0137634.s001.tif]

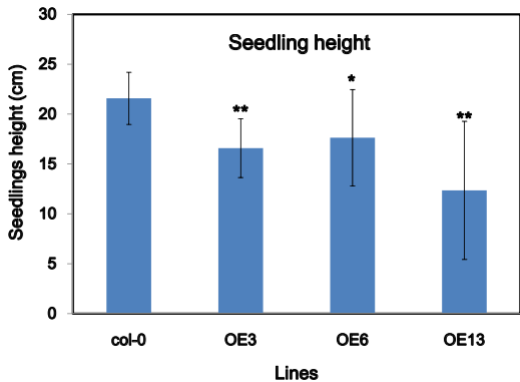

Supplement: S2 Fig — Arabidopsis seedlings of col-0 and HbCBF1 overexpression lines OE3, OE6 and OE13 were cultured as described in Material and methods. The seedling height was measured at 30 days old. The data were presented as mean ± standard error of mean of 15 to 20 plants. Star symbol (*) shows the significance when compared with col-0 plants. The statistical significance analysis was performed by using the Student’s t-test, and p < 0.05 was recognized as significant. (PDF) [file pone.0137634.s002.pdf]

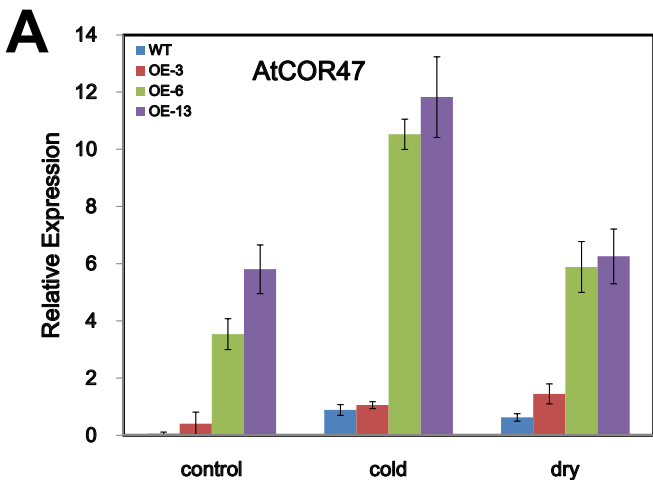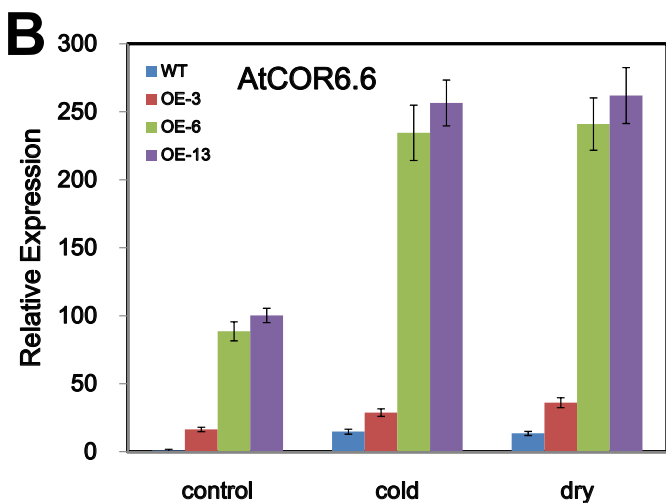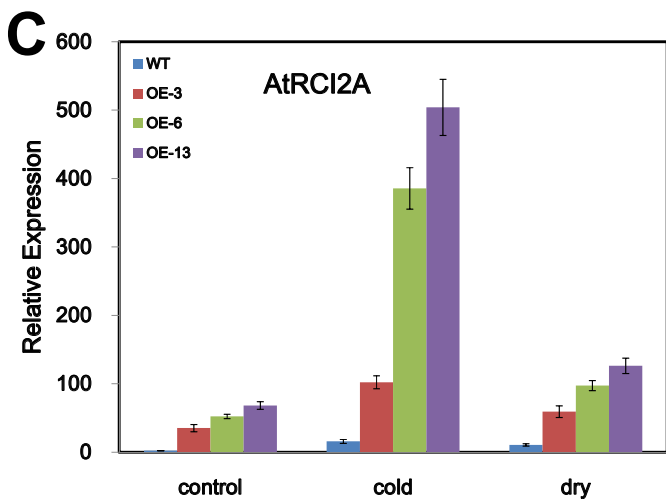

Supplement: S3 Fig — AtCOR47 (A), AtCOR6.6 (B) and AtRCI2a (C) expression in col-0, OE-3, OE-6 and OE-13 plants under nonacclimated (control), 5-day cold-acclimated (cold) or 10-days drought stress (drought). Data were presented as mean ± standard error of mean of three biological replicates. Significance was determined by Student’s t test at the probability levels of P < 0.05. (PDF) [file pone.0137634.s003.pdf]
