# Supplementary material for: Functional Characterization of Hevea brasiliensis CRT/DRE Binding Factor 1 Gene Revealed Regulation Potential in the CBF Pathway of Tropical Perennial Tree
Source: PLoS One. 2015 Sep 11;10(9):e0137634. doi: 10.1371/journal.pone.0137634 (PMC4567348; doi:10.1371/journal.pone.0137634)
Supplement: S1 Table — (DOCX) [file pone.0137634.s004.docx]

**Supplementary Table S1. Oligo used in this study**

| Oligo | sequence |
| --- | --- |
| C007 | CAYCCNATHTAYMGNGGNGT |
| C008 | GGNRRNARCATNCCYTCNGCC |
| C010 | TCTTGATTTCTTGTTGGGTTCT |
| C011 | ACGACAGAGTCAGCGCCGGA |
| C013 | AGGAACTCGGGAAGTGGGT |
| C014 | CCAGTAGCCCGGCATTGCA |
| Pro_C020 | CCCTCGGATCCATGGATGTTTTC |
| Pro_C021 | CAAGCTATGCGGCCGCTCTTAT |
| COR15a | TTTCATGGCCGACCTGCTTTTTT |
| COR15b | CTGATGGCCGACCTCTTTTTT |
| RD29a | ATATACTACCGACATGAGTTCT |
| RD29b | CTACCGACATGAGTTCCAAAAAA |
| M1 | TTTCAAGAATTCACTGCTTTTTT |
| M2 | TTTCATGGTATGTCTGCTTTTTT |
| M3 | TTTCATGGAATCACTGCTTTTTT |
| HbOE_XbaI | CCTCTAGACTATGGATGTTTTCCCT |
| HbOE_SacI | CCCGAGCTCTTATATAGAAAAACTCCA |
| Hb18SS_1 | AAGCAAGCCTACGCTCTGG |
| Hb18SA_2 | GCTCCACCAACTAAGAACGG |
| ACTIN7-F | GTCCCTGCCATGTATGTT |
| ACTIN7-A | CCCGCAAGATCAAGACG |
| HbCBF1-f | CAATGCCAGCTTCTTCATAAAC |
| HbCBF1-A | CCCATACAACATCTCTTTCAATC |
| RD29A-F | TTGCCGAGAAACTTCAGATTG |
| RD29A-A | ACCACCGAACCATCCTTTA |
| COR15A-F | ATCTACGCCGCTAAAGG |
| COR15A-A | AATGTATCTGCGGTTTCACT |
| AtCBF1-f | GTGATACGACGACCACG |
| AtCBF1-A | AACAAAGTCGGCATCCC |
| AtCBF2-F | GAGGCTATTTATACGCCGGA |
| AtCBF2-A | CATAAGGACACGTCATCATCTC |
| AtCBF3-F | GCTATTTACACGGCGGAAC |
| AtCBF3-A | CCATAACGATACGTCGTCA |
| AtCOR47-F | GTTGTTATTGTGGCTTTCGTT |
| AtCOR47-A | GAGTATACGATGAGTGTTATGGG |
| AtCOR6.6-F | AAAGAGTATATCGGATGCGG |
| AtCOR6.6-A | ACAAGTACGATGAGTACGAGA |
| AtRCI2A-F | AATGAGTACAGCTACTTTCGTT |
| AtRCI2A-A | TAAATGGCGTATATGATCCCAGG |
